# Protocol for an economic evaluation alongside the Re-Evaluating the Inhibition of Stress Erosions (E-REVISE) trial

**DOI:** 10.1136/bmjopen-2025-106841

**Published:** 2025-11-09

**Authors:** Brittany Humphries, Nicole Zytaruk, Diane Heels-Ansdell, Vincent Lau, Bram Rochwerg, Robert Fowler, Yifan Yao, Deborah J Cook, Feng Xie

**Affiliations:** 1 Health Research Methods, Evidence and Impact, McMaster University, Hamilton, Ontario, Canada; 2 Critical Care Medicine, University of Alberta, Edmonton, Alberta, Canada; 3 Medicine, McMaster University, Hamilton, Ontario, Canada; 4 Interdepartmental Division of Critical Care, University of Toronto, Toronto, Ontario, Canada; 5 Centre for Health Economics and Policy Analysis, McMaster University, Hamilton, Ontario, Canada

**Keywords:** INTENSIVE & CRITICAL CARE, HEALTH ECONOMICS, GASTROENTEROLOGY

## Abstract

**Abstract:**

**Introduction:**

Economic evaluations in healthcare can guide practice and inform policy. The objective of this paper is to present the protocol for a health economic evaluation comparing the cost-effectiveness of prophylactic treatment using pantoprazole 40 mg daily compared with no pantoprazole to prevent upper gastrointestinal (GI) bleed among invasively ventilated patients.

**Methods and analysis:**

This is an economic evaluation conducted alongside the *R*e-*E*valuating the *I*nhibition of *S*tress *E*rosions (REVISE) trial (ClinicalTrials.gov NCT03374800). The primary outcome is the incremental cost per clinically important upper GI bleed prevented. The base-case analysis will focus on the entire international cohort of 4821 REVISE patients. The analysis will be conducted from a healthcare payer perspective over a time horizon of ICU admission to hospital discharge or death. To facilitate comparisons across countries given the international scope of the REVISE trial, costs will be presented in United States dollars. The study protocol was developed following the Professional Society for Health Economics and Outcomes Research guidelines.

**Ethics and dissemination:**

The trial was approved by each participating institution; this economic evaluation was approved by the Hamilton Integrated Research Ethics Board. Given widespread daily use of proton pump inhibitors for critically ill patients, the results of this economic evaluation will be of high relevance to patients, family members, physicians, pharmacists, policymakers and guideline developers. Integrated knowledge translation will involve periodic progress reports to collaborators. End-of-study knowledge translation will include rounds, videoconferences, abstracts and slide-decks for intensive care unit quality councils and healthcare organisations, and open-access publications. Patient and family partners will co-create lay language summaries for traditional and social media to help inform all interest groups.

**STRENGTHS AND LIMITATIONS OF THIS STUDY**

- Conducting an economic evaluation alongside a randomised clinical trial enhances internal validity.
- The use of individual level patient data from 4821 participants allows for detailed and precise cost and outcome estimates.
- The short time horizon and limited hospital length of stay may underestimate long term costs and benefits.
- The absence of patient-reported outcome measures prevents estimation of cost per quality-adjusted life year.

## Background

 Critically ill patients who require life support may develop stress erosions and ulceration in the proximal gastrointestinal (GI) tract which can cause upper GI bleeding. This occurs in up to 4% of patients in the intensive care unit (ICU) without prophylaxis.^1 2^ Acid suppression in the form of proton pump inhibitors (PPIs) is routinely administered to critically ill patients to prevent upper GI bleeding from stress-induced ulceration.^3^ However, some studies suggest that potential adverse effects such as infection in the lungs (pneumonia) or bowels (*Clostridioides difficile*) may be more common than GI bleeding or associated with greater morbidity, mortality and costs.^4^ As a result, recent guidelines issue conditional recommendations for PPIs in critically ill patients at high risk of bleeding based on the moderate certainty evidence.^5^

To address this gap in understanding, our team conducted the *R*e-*E*valuating the *I*nhibition of *S*tress *E*rosions (REVISE) trial^6^ in which 4821 critically ill adults who were invasively mechanically ventilated were allocated to receive intravenous pantoprazole 40 mg daily or matching placebo. Clinically important upper GI bleeding was reduced with pantoprazole, occurring in 1.0% patients receiving pantoprazole and in 3.5% receiving placebo (HR, 0.30; 95% CI), 0.19–0.47). Death at 90 days was similar in the two groups (29.1% in pantoprazole group and 30.9% in placebo group; HR, 0.94; 95% CI, 0.85–1.04). Patient-important GI bleeding was also reduced with pantoprazole. There were no significant differences in other key secondary trial outcomes such as ventilator-associated pneumonia, *Clostridioides difficile* infection or length of hospital stay.

In addition to investigating the clinical outcomes associated with the use of PPIs, it is also important to understand the economic implications of stress ulcer prophylaxis. Health economic evaluations compare two or more healthcare interventions with respect to their costs (eg, resource utilisation) and effects (eg, health outcomes).^7^ Evaluating cost and resource use alongside the REVISE trial provides a unique opportunity to gain insight into the net effect of an intervention. The ICU is one of the most costly settings to care for patients, accounting for 20%–50% of all hospital costs in the USA.^8 9^ Every dollar spent—particularly within this high-resource environment—has an opportunity cost. The cost of acid suppression in the ICU is often misunderstood because of the low acquisition cost of pantoprazole per dose.

## Objective

The objective of this paper is to report the protocol of estimating the cost-effectiveness of prophylactic pantoprazole 40 mg intravenously daily compared with no pantoprazole, determined by the incremental cost to prevent a clinically important upper GI bleed among invasively ventilated patients.

## Methods

### Overview of REVISE

REVISE is an investigator-initiated, international, randomised, blinded, controlled trial (ClinicalTrials.gov NCT03374800). Patients aged >18 years who were admitted to the ICU and expected to remain invasively ventilated beyond the calendar day after randomisation were randomised to receive either placebo (0.9% sodium chloride) or pantoprazole 40 mg intravenously daily. Randomisation was stratified by study centre and use of prehospital acid suppression. All other aspects of care were at the discretion of the treating team, including basic and advanced life support, enteral nutrition and other interventions to prevent ICU-acquired complications. The primary efficacy outcome of the trial was clinically important upper GI bleeding within 90 days and the primary safety outcome was 90-day all-cause mortality. Secondary trial outcomes included ventilator-associated pneumonia, *Clostridioides difficile* infection and patient-important upper GI bleeding. The REVISE protocol^6^ and results^1^ are reported separately.

### E-REVISE design

**Figure 1:**
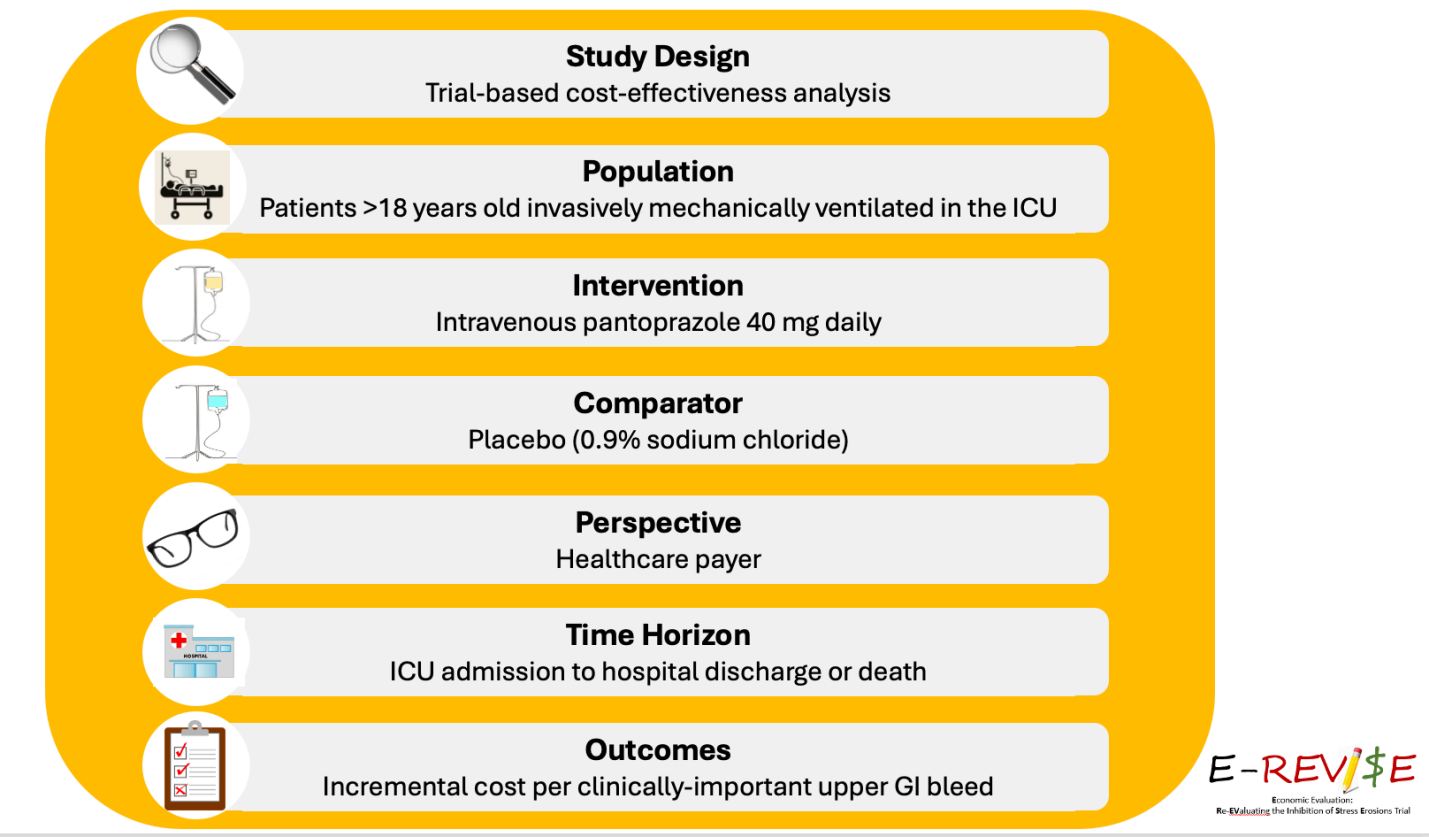
E-REVISE framework. A figure summarising the key considerations for the design of the cost-effectiveness analysis. E-REVISE, Re-Evaluating the Inhibition of Stress Erosions; ICU, intensive care unit;

This is an economic evaluation conducted alongside the REVISE trial (E-REVISE). The purpose of E-REVISE is to estimate the incremental cost-effectiveness of prophylactic pantoprazole 40 mg intravenously compared with no pantoprazole to prevent GI bleeding among mechanically ventilated patients. The primary outcome is the incremental cost per clinically important upper GI bleed prevented. The analysis will be conducted from a healthcare payer’s perspective over a time horizon of ICU admission to hospital discharge or death. The study protocol was developed following the good practice guidelines of Canada’s Drug Agency^10^ and the Professional Society for Health Economics and Outcomes Research.^11^ A summary of the study framework is presented in figure 1.

### Study sample

The REVISE trial recruited 4821 patients from 68 study centres across Australia, Brazil, Canada, United Kingdom, Kuwait, Pakistan, Saudi Arabia and the USA. The sample size calculation, which was designed to detect a statistically significant absolute difference in the primary efficacy outcome (ie, clinically important upper GI bleeding), is described elsewhere.^6^ This base-case analysis will focus on the entire international cohort of 4821 REVISE patients.

### Data sources

We will use patient-level data collected in the REVISE trial to obtain information describing patients’ socio-demographic and clinical characteristics (eg, age, sex, comorbidities, prehospital hospital acid suppression, Acute Physiology and Chronic Health Evaluation (APACHE) II score, ICU admitting diagnosis, SARS-CoV-2 status), clinical outcomes (eg, GI bleeding, ventilator-associated pneumonia, *Clostridioides difficile* infection, death), and healthcare resource utilisation (eg, drug administration, laboratory and diagnostic tests, packed red blood cells and other blood product administration, procedures, nutrition, surgeries, advanced life support strategies, number of days in hospital and ICU). As the majority of patients in REVISE were recruited from Canadian study sites (*n*=3265/4821, 68%), unit costs for each resource item in the base case will be obtained from publicly available sources in Canada, such as the Canadian Institute for Health Information,^12^ Ontario Drug Benefit Formulary,^13^ the Schedule of Benefits for Laboratory Services,^14^ and the Schedule of Benefits for Physician Services,^15^ Canadian Blood Services,^16^ as well as recently completed critical care health economic evaluations.^17 18^ We will obtain additional information from wholesaler and distributors’ catalogues to address any outstanding data gaps (eg, if data on a drug’s unit cost is not available publicly). If multiple unit costs are available, we will take the median to account for the variability of costs within and across jurisdictions. Only in-hospital costs incurred from the healthcare payer perspective will be considered. For patients enrolled in other countries, their resource use will be estimated using the data collected in the REVISE trial. The unit costs will be based on the Canadian unit costs as a proxy.

### Statistical analyses

Descriptive statistics (mean, standard-deviation, frequency, percentage) will be used to summarise patients’ baseline characteristics, clinical outcomes and costs. While medians will be used at the costing level, mean costs will be presented at the reporting level because they are more informative for resource allocation decisions. The resources used will be multiplied by their unit costs and then summed to calculate individual patient costs. In the base-case analysis, the incremental cost-effectiveness ratio (ICER) will be estimated by dividing the incremental cost by the incremental effect for pantoprazole and no pantoprazole groups. Incremental effects are defined as the difference in per-patient event rates or the difference in the proportion experiencing a clinical event between groups. The primary outcome is the incremental cost per clinically important upper GI bleed prevented. ICERs will not be calculated in situations where one treatment strategy is both less costly and more effective (ie, dominant) according to the clinical outcome considered.

The base case analyses will be conducted according to the intention-to-treat principle as the REVISE trial had a low rate of missing data (ie, 15 patients missing data on ICU stay data and 17 missing data on hospital stay). All costs will be converted to 2025 values using the Canadian Consumer Price Index. To facilitate comparisons across countries, costs will be presented in United States dollars using a mean exchange rate (1 CAD$=0.714 USD$ in August 2025). No discounting will be applied due to the short time horizon (< 1 year). The analyses will be conducted using R (V.4.4.0) statistical software.

### Sensitivity analyses

Sensitivity analyses will be conducted to account for potential differences in patient characteristics, resource use and unit costs across countries and outside of controlled clinical trials.^19^ First, we will conduct sensitivity analyses using unit costs from the United States for key resource use items (eg, pantoprazole intravenous or oral, ICU and hospital stay). Second, we will conduct an analysis restricted to the 3265 Canadian patients enrolled in REVISE.

To address sampling uncertainty, we will perform a probabilistic sensitivity analysis using non-parametric bootstrapping techniques to generate 95% confidence intervals (CIs).^11^ One-way sensitivity analyses will be conducted for key influential variables (eg, cost per day cost of care in ICU) and assumptions (eg, cost of an upper gastrointestinal bleeding event) to assess their impact on observed ICERs in terms of direction and magnitude of effect.

We will also conduct an additional sensitivity analysis to address missing resource use data on ICU and hospital stay using multiple imputation.^20^ Missing data will be reviewed to assess whether they are missing completely at random. An appropriate imputation method will then be selected according to the type and distribution of the missing data.^20^

### Subgroup analyses

As with other REVISE trial analyses, we will conduct subgroup analyses according to: (1) use of prehospital acid suppression (proton pump inhibitors or histamine-2-receptor antagonists versus no prehospital acid suppression); (2) APACHE II score (≥25 vs <25); (3) ICU admitting diagnosis (medical vs surgical/trauma); (4) SARS-CoV-2 status (positive vs negative); and (5) sex (female vs male).^6^

### Patient and public involvement

In a mixed-methods study,^21^ patients and family members developed a secondary REVISE trial outcome of patient-important upper gastrointestinal bleeding^22^ which in the REVISE trial was also found to be reduced in those receiving prophylactic pantoprazole. Regarding the design and conduct of this economic evaluation, members of the public were not involved; however, when the economic analysis is complete, we will co-create knowledge translation interventions, involving them in the dissemination plans.

### Ethics and dissemination

All participating centres received research ethics approval before initiation by hospital, region or country, including, but not limited to Australia: Northern Sydney Local Health District Human Research Ethics Committee and Mater Misericordiae Ltd Human Research Ethics Committee; Brazil: Comissão Nacional de Ética em Pesquisa; Canada: Hamilton Integrated Research Ethics Board; Kuwait: Ministry of Health Standing Committee for Coordination of Health and Medical Research; Pakistan: Maroof Institutional Review Board; Saudi Arabia: Ministry of National Guard Health Affairs Institutional Review Board: United Kingdom: Hampshire B Research Ethics Committee; United States: Institutional Review Board of the Nebraska Medical Centre. This economic evaluation has received approval by the lead Research Ethics Board - Hamilton Integrated Research Ethics Board (REB) (Project Number: 19132; 20 August 2025).

We will follow the Consolidated Health Economic Evaluation Reporting Standards 2022 (CHEERS 2022) Statement^23^ to report the study findings. Given universal daily stress ulcer prophylaxis prescribing, the results will be of high relevance to physicians, pharmacy departments, patients and policy makers in Ontario and elsewhere. Integrated knowledge translation will involve progress reports at thrice yearly ICU and health economic fora. End-of-study knowledge translation will include rounds, videoconferences, abstracts and slide decks for ICU quality councils and healthcare organisations, and open-access publications. Patient and family partners will co-create lay language summaries for traditional and social media to help inform all interested constituent groups.

## Discussion

E-REVISE is proposed in the context of a living, learning, healthcare system responsible for stewardship of scarce health resources. The REVISE trial is the largest trial undertaken comparing pantoprazole versus placebo for critically ill patients. E-REVISE will address the cost-effectiveness of pantoprazole for stress ulcer prophylaxis in ICU, to inform physicians, pharmacists, policymakers and the general public about whether the cost provides value for their healthcare dollar. This lifecycle approach to health technology assessment in E-REVISE is innovative in the contexts of both critical care and health economics, where attention is typically focused on new drugs or devices rather than interventions in common use.^24^

### Generalisability

REVISE is an international trial and it is possible that resource use and unit costs could vary between (and within) countries. However, from a clinical perspective, management of a clinically important upper GI is unlikely to differ substantially between jurisdictions as there are only a limited number of tests (eg, haemoglobin measurement, diagnostic endoscopy) and treatments (eg, intravenous pantoprazole, blood transfusions, therapeutic endoscopy) that are commonly used. From an economic perspective, the modest number of enrolled patients in some countries in REVISE makes it difficult to consider country in multilevel modelling.^25^ In addition, costs typically have substantial variations (eg, some patients have more expensive care than others, and some have complications while others have none). As a result, traditional statistical methods may not be appropriate. Instead of investigating statistical significance, economic evaluations such as ours usually focus on reporting mean costs and their exploring uncertainty using probabilistic and other sensitivity analyses. As part of this, we plan to conduct a sensitivity analysis comparing the results among Canadian patients with the full study sample to assess the magnitude of difference between jurisdictions.^26^ In addition, to support the interpretation of results across countries, the base case will be reported in United States dollars and resource units will be provided so that decision-makers can apply their own local unit costs.

### Strengths and limitations

This health economic evaluation has certain strengths, including measurement of clinical and economic data alongside a randomised clinical trial. This offers several advantages, such as the effect of randomisation to help ensure comparable baseline characteristics between groups. In addition, a comprehensive collection of clinical and economic data within a trial reduces data collection time and costs, especially for data that are impractical to obtain retrospectively.^19^ Timely economic data can be useful to healthcare policymakers locally and at the level of jurisdictional health services delivery to aid budgetary and healthcare resource allocation decisions. This is especially true if the intervention being evaluated is already in current practice or is the current standard of care, such is the case with pantoprazole.

This economic evaluation also has some limitations. One limitation, which is common in trials, is that the same treatment effects and costs may not be observed in routine clinical practice. This may limit the generalisability of findings. To reduce the potential for investigator bias, particularly as the REVISE results have been published,^1^ we have prespecified the parameters for the analysis (eg, subgroup and sensitivity analyses); furthermore, analysts conducting this health economic analysis will be blinded to study drug until the final analysis is complete. Another limitation is the short time horizon of the analysis and lack of out-patient follow-up as additional costs incurred after discharge by patients and their caregivers will not be included. While the trial recorded where patients went after discharge (eg, acute care facility, home care, long term care), these downstream costs are out of scope of the current analysis. Only a public healthcare payer perspective was considered to align with Canadian HTA recommendations.^10^ The REVISE case report forms were designed to collect key information for the randomised trial; as such, some data relevant to this health economic evaluation need to be derived from other sources (eg, dosage of certain medications). Finally, REVISE was designed to examine the clinical effect of the prophylactic drug on upper gastrointestinal bleeding. Willingness-to-pay thresholds for this outcome have not previously been developed, in distinction to outcomes such as the incremental cost per life, life-year or quality-adjusted life-year gained. Therefore, our results may need to be interpreted in comparison with the clinical outcomes and costs of other interventions, and by patients, providers and policy makers to assess relative value.

In summary, E-REVISE will complement the REVISE trial with a prespecified prospective comprehensive economic evaluation. Cost-effectiveness analyses can aid in clinical guideline development and be useful to healthcare policymakers to aid in resource allocation decisions.
