# Supplementary material for: Protocol for an economic evaluation alongside the Re-Evaluating the Inhibition of Stress Erosions (E-REVISE) trial
Source: BMJ Open. 2025 Nov 9;15(11):e106841. doi: 10.1136/bmjopen-2025-106841 (PMC12658520; doi:10.1136/bmjopen-2025-106841)
Supplement: Supplementary file 1 [file bmjopen-2025-106841.reviewer_comments.pdf]

PEER REVIEW HISTORY

BMJ Open publishes all reviews undertaken for accepted manuscripts. Reviewers are asked to complete a checklist review form and are provided with free text boxes to elaborate on their assessment. These free text comments are reproduced below.

ARTICLE DETAILS

Title (Provisional)

Protocol for an Economic Evaluation Alongside the Re-Evaluating the Inhibition of Stress Erosions (E-REVISE) Trial

Authors

Humphries, Brittany; Zytaruk, Nicole; Heels-Ansdell, Diane; Lau, Vincent Issac; Rochwerg, Bram; Fowler, Robert; Yao, Yifan; Cook, Deborah J; Xie, Feng

---

VERSION 1 - REVIEW

**Reviewer**	**1**
**Name**	**Gc, Vijay**
**Affiliation**	**University of York, Centre for Health Economics**
**Date**	**20-Aug-2025**
**COI**	

This is a clearly written health economics protocol outlining the economic evaluation alongside the REVISE trial. Below are the comments and suggestions related to clarifications and refinements aimed at enhancing the transparency, validity, and applicability of the proposed analysis.

1) The background needs strengthening as the statement of the problem is not clearly defined. For example, include the incidence or prevalence of the condition.

2) Given the nature of the multicounty trial, please specify whether the economic burden, i.e., 20-25% of all hospital costs, is for Canada or another setting.

3) As REVISE is a multinational study, it was not clear whether the analysis would only include patients recruited from study centres in Canada. The authors mention on page 9 that the base case analysis will include patients recruited from Canadian sites, but don't specify this for resource use data (page 10).

4) It is expected that there is variation in the clinical practice/management between countries, and so is the case with the resource usage and unit costs. It is unclear whether the authors plan to use country-specific unit costs (and then convert them to CAD$) in the sensitivity analyses. Please also discuss the issue around between-location variability in cost-

effectiveness and the generalisability of the cost-effectiveness results. The authors may find doi:10.1002/hec.914, doi:10.1007/s40273-015-0371-y useful.

5) The statistical analyses section is brief. It needs to include a description of the proposed methods for analysing cost and outcome data, for example, the use of regression models to analyse costs and outcome data, covariate adjustment.

Given the above comments, the strengths and limitations section may need to be revised.

6) Fig. 1, please make sure all the texts inside the boxes are readable.

VERSION 1 - AUTHOR RESPONSE

Reviewer: 1
Dr. Vijay Gc, University of York
Comments to the Author:
This is a clearly written health economics protocol outlining the economic evaluation alongside the REVISE trial. Below are the comments and suggestions related to clarifications and refinements aimed at enhancing the transparency, validity, and applicability of the proposed analysis.

** Thank you for your time peer reviewing our protocol. We appreciate it.

1) The background needs strengthening as the statement of the problem is not clearly defined. For example, include the incidence or prevalence of the condition.

** We appreciate this suggestion and have adjusted the background accordingly.

First, we have added more information on the condition. Excerpted here: "*Critically ill patients who require life support may develop stress erosions and ulceration in the proximal gastrointestinal (GI) tract which can cause upper GI bleeding. This occurs in up to 4% of patients in the intensive care unit (ICU) without prophylaxis. [1,2] Acid suppression in the form of proton pump inhibitors (PPIs) is routinely administered to critically ill patients to prevent upper GI bleeding from stress-induced ulceration.[3]*"

Second, we have more clearly stated the problem from an economic perspective. Excerpted here: "*In addition to investigating the clinical outcomes associated with the use of PPIs, it is also important to understand the economic implications of stress ulcer prophylaxis. Health economic evaluations compare two or more healthcare interventions with respect to their costs (e.g., resource utilization) and effects (e.g., health outcomes).[7] Evaluating cost and resource use alongside the REVISE trial provides a unique opportunity to gain insight into the net effect of this intervention.*"

2) Given the nature of the multicounty trial, please specify whether the economic burden, i.e., 20-25% of all hospital costs, is for Canada or another setting.

** Thank you for pointing this out. We agree that the text was vague and have modified it accordingly. We now clarify that these references refer to economic burden in the US context.

Excerpted here: "*The intensive care unit (ICU) is one of the most costly settings to care for patients, accounting for 20-50% of all hospital costs in the United States.[8, 9]*"

3) As REVISE is a multinational study, it was not clear whether the analysis would only include patients recruited from study centres in Canada. The authors mention on page 9 that the base case analysis will include patients recruited from Canadian sites, but don't specify this for resource use data (page 10).

** We appreciate the chance to provide additional details. We are actually using all 4,821 international patients in the trial to contribute to the base case analysis, but Canadian unit costs will be used for the base case analysis, converted into US dollars (that is a clarification from our originally submitted protocol, so thank you for this question). There will be a sensitivity analysis considering only Canadian patients and only Canadian costs. Our methods are expanded and clarified accordingly.

Excerpted here under 'Study sample': "*The REVISE trial recruited 4,821 patients from 68 study centres across Australia, Brazil, Canada, United Kingdom, Kuwait, Pakistan, Saudi Arabia, and the United States. The sample size calculation, which was designed to detect a statistically significant absolute difference in the primary efficacy outcome (i.e., clinically-important upper GI bleeding), is described elsewhere.[6] This base-case analysis will focus on the entire international cohort of 4,821 REVISE patients*."

Excerpted here under 'Data sources': "*As the majority of patients in REVISE were recruited from Canadian study sites (n= 3,265/4,821, 68%), unit costs for each resource item in the base case will be obtained from publicly available sources in Canada, such as the Canadian Institutes for Health Information,[12] Ontario Drug Benefit Formulary,[13] the Schedule of Benefits for Laboratory Services,[14] and the Schedule of Benefits for Physician Services,[15] Canadian Blood Services,[16] as well as recently completed critical care health economic evaluations.[17, 18]*"

Excerpted here under 'Sensitivity analyses': "*Second, we will conduct an analysis restricted to the 3,265 Canadian patients enrolled in REVISE.*"

4) It is expected that there is variation in the clinical practice/management between countries, and so is the case with the resource usage and unit costs. It is unclear whether the authors plan to use country-specific unit costs (and then convert them to CAD$) in the sensitivity analyses. Please also discuss the issue around between-location variability in cost-effectiveness and the generalisability of the cost-effectiveness results. The authors may find doi:10.1002/hec.914, doi:10.1007/s40273-015-0371-y useful.

Given the above comments, the strengths and limitations section may need to be revised.

** Thank you for pointing this out. Instead of modifying the strengths and limitations, we have added a new section on generalizability to explicitly address this very issue from both clinical and economic perspectives. We have also reviewed and incorporated the two references you shared:

Excerpted here under 'Generalizability': *"REVISE is an international trial and it is possible that resource use and unit costs could vary between (and within) countries. However, from a clinical perspective, management of a clinically important upper GI is unlikely to differ substantially between jurisdictions as there are only a limited number of tests (e.g., hemoglobin measurement, diagnostic endoscopy) and treatments (e.g., intravenous pantoprazole, blood transfusions, therapeutic*

endoscopy) that are commonly used. From an economic perspective, the modest number of enrolled patients in some countries in REVISE make it difficult to consider country in multilevel modeling.[25] In addition, costs typically have substantial variations (e.g., some patients have more expensive care than others, and some have complications while others none). As a result, traditional statistical methods may not be appropriate. Instead of investigating statistical significance, economic evaluations such as ours usually focus on reporting mean costs and their exploring uncertainty using probabilistic and other sensitivity analyses. As part of this, we plan to conduct a sensitivity analysis comparing the results among Canadian patients with the full study sample to assess the magnitude of difference between jurisdictions.[26] In addition, to support the interpretation of results across countries, the base case will be reported in United States dollars and resource units will be provided so that decision-makers can apply their own local unit costs."

5**) The statistical analyses section is brief. It needs to include a description of the proposed methods for analysing cost and outcome data, for example, the use of regression models to analyse costs and outcome data, covariate adjustment.**

** As discussed above, we did not use multilevel modelling or regression models. However, we welcome the opportunity to expand on the statistical analysis, which we have done throughout the Methods section.

Excerpted here under 'Data sources': "*We will use patient-level data collected in the REVISE trial to obtain information describing patients' socio-demographic and clinical characteristics (e.g., age, sex, comorbidities, pre-hospital hospital acid suppression, Acute Physiology and Chronic Health Evaluation (APACHE) II score, ICU admitting diagnosis, SARS-CoV-2 status), clinical outcomes (e.g., GI bleeding, ventilator-associated pneumonia, Clostridioides difficile infection, death), and healthcare resource utilization (e.g., drug administration, laboratory and diagnostic tests, packed red blood cells and other blood product administration, procedures, nutrition, surgeries, advanced life support strategies, number of days in hospital and ICU). As the majority of patients in REVISE were recruited from Canadian study sites (n= 3,265/4,821, 68%), unit costs for each resource item in the base case will be obtained from publicly available sources in Canada, such as the Canadian Institutes for Health Information,[12] Ontario Drug Benefit Formulary,[13] the Schedule of Benefits for Laboratory Services,[14] and the Schedule of Benefits for Physician Services,[15] Canadian Blood Services,[16] as well as recently completed critical care health economic evaluations.[17, 18] We will obtain additional information from wholesaler and distributors' catalogues to address any outstanding data gaps (e.g., if data on a drug's unit cost is not available publicly). If multiple unit costs are available, we will take the median to account for the variability of costs within and across jurisdictions. Only in-hospital costs incurred from the healthcare payer perspective will be considered. For patients enrolled in other countries, their resource use will be estimated using the data collected in the REVISE trial. The unit costs will be based on the Canadian unit costs as a proxy*."

Excerpted here under 'Statistical analyses': "*Descriptive statistics (mean, standard deviation, frequency, percentage) will be used to summarize patients' baseline characteristics, clinical outcomes, and costs. While medians will be used at the costing level, mean costs will be presented at the reporting level because they are more informative for resource allocation decisions. The resources used will be multiplied by their unit costs and then summed to calculate individual patient costs. In the base-case analysis, the incremental cost-effectiveness ratio (ICER) will be estimated by dividing*

the incremental cost by the incremental effect for pantoprazole and no pantoprazole groups. Incremental effects are defined as the difference in per-patient event rates or the difference in the proportion experiencing a clinical event between groups. The primary outcome is the incremental cost per clinically-important upper GI bleed prevented. ICERs will not be calculated in situations where one treatment strategy is both less costly and more effective (i.e., dominant) according to the clinical outcome considered.

The base case analyses will be conducted according to the intention-to-treat principle as the REVISE trial had a low rate of missing data (i.e. 15 patients missing data on ICU stay data and 17 missing data on hospital stay). All costs will be converted to 2025 values using the Canadian Consumer Price Index. To facilitate comparisons across countries, they will be presented in United States dollars using a mean exchange rate (1 CAD$=0.714 USD$ in August 2025). No discounting will be applied due to the short time horizon (< 1 year). The analyses will be conducted using R (version 4.4.0) statistical software."

Excerpted here under 'Sensitivity analyses: "*Sensitivity analyses will be conducted to account for potential differences in patient characteristics, resource use, and unit costs across countries and outside of controlled clinical trials.[19] First, we will conduct sensitivity analyses using unit costs from the United States for key resource use items (e.g., pantoprazole intravenous or oral, ICU, and hospital stay). Second, we will conduct an analysis restricted to the 3,265 Canadian patients enrolled in REVISE.*

To address sampling uncertainty, we will perform a probabilistic sensitivity analysis using non-parametric bootstrapping techniques to generate 95% confidence intervals (CIs).[11] One-way sensitivity analyses will be conducted for key influential variables (e.g., cost per day cost of care in ICU) and assumptions (e.g., cost of an upper gastrointestinal bleeding event) to assess their impact on observed ICERs in terms of direction and magnitude of effect.

We will also conduct an additional sensitivity analysis to address missing resource use data on ICU and hospital stay using multiple imputation.[20] Missing data will be reviewed to assess whether they are missing completely at random. An appropriate imputation method will then be selected according to the type and distribution of the missing data.[20]"

6) Fig. 1, please make sure all the texts inside the boxes are readable.

** We have done so, thanks.

Additional modifications by authors:

1) We have deleted the word 'stakeholder' from the manuscript due to concerns regarding colonialism connotations. It has been replaced with phrases such as 'interest groups' or 'constituent interest groups' etc.

Excerpted here under 'Abstract': "*Patient and Family Partners will co-create lay language summaries for traditional and social media to help inform all interest groups*."

Excerpted here under 'Ethics and dissemination': "*Patient and Family Partners will co-create lay language summaries for traditional and social media to help inform all interested constituent groups.*"

2) We have focused on our primary objective and primary outcome, omitting secondary exploratory analyses.

3) We added another citation to refer to recent critical care economic evaluations:

Lau VI, Fowler R, Pinto R, Tremblay A, Borgia S, Carrier FM, Cheng MP, Conly J, Costiniuk CT, Daley P, Duan E, Durand M, Fontela PS, Farjou G, Fralick M, Geagea A, Grant J, Keynan Y, Khwaja K, Lee N, Lee TC, Lim R, O'Neil CR, Papenburg J, Semret M, Silverman M, Sligl W, Somayaji R, Tan DHS, Tsang JLY, Weatherald J, Yansouni CP, Zarychanski R, Murthy S; E-CATCO authors, AMMI Clinical Research Network and Canadian Critical Care Trials Group. Cost-effectiveness of remdesivir plus usual care versus usual care alone for hospitalized patients with COVID-19: an economic evaluation as part of the Canadian Treatments for COVID-19 (CATCO) randomized clinical trial. CMAJ Open. 2022 Sep 6;10(3):E807-E817. doi: 10.9778/cmajo.20220077. PMID: 36199248; PMCID: PMC9477473.

4) We have also made minor revisions to improve the manuscript.

Excerpted here under 'Discussion: "*REVISE is proposed in the context of a living, learning, healthcare system responsible for stewardship of scarce health resources. The REVISE trial is the largest trial undertaken comparing pantoprazole versus placebo for critically ill patients. E-REVISE will address the cost-effectiveness of pantoprazole for stress ulcer prophylaxis in ICU, to inform physicians, pharmacists, policymakers and the general public about whether the cost provides value for their healthcare dollar. This lifecycle approach to health technology assessment in E-REVISE is innovative in the contexts of both critical care and health economics, where attention is typically focused on new drugs or devices rather than interventions in common use.[23]*"
